# ^1^H NMR for Comparative Metabolic Analysis of Whey and WPC-80

**DOI:** 10.3390/metabo15120770

**Published:** 2025-11-28

**Authors:** Ingrid Sousa, Gaia Meoni, Leonardo Tenori, Marta Pozza, Massimo De Marchi, Giovanni Niero

**Affiliations:** 1 Department of Agronomy, Food, Natural Resources, Animals and Environment, University of Padua, 35020 Legnaro, Italy; ingrid.sousa@unipd.it (I.S.);; 2 Department of Chemistry “Ugo Schiff”, University of Florence, 50121 Sesto Fiorentino, Italy

**Keywords:** whey, whey concentrate, metabolome, metabolomics

## Abstract

**Background/Objectives:** Metabolites are low-molecular-weight organic compounds (<1 kDa) that act as intermediates and end products of cellular metabolism. Their characterization provides valuable information on the nutritional quality, functionality, and potential health impacts of food products. In the dairy sector, proton nuclear magnetic resonance (^1^H NMR) spectroscopy has emerged as a powerful tool for metabolite profiling, enabling the simultaneous identification and quantification of diverse compounds. In this study, ^1^H NMR was applied to characterize and compare the metabolic composition of whey, a major by-product of cheese and yogurt production, and whey protein concentrate (WPC-80), a whey derivative containing approximately 80% protein by weight and rich in essential amino acids. **Methods:** Five whey and four WPC-80 samples from a single Parmigiano Reggiano dairy plant were collected, each representing a biologically independent sample. Statistical evaluation was performed using Mann–Whitney U tests to identify significantly different metabolites between groups, while principal component analysis and partial least squares discriminant analysis were employed to assess group separation and determine discriminant metabolites. **Results:** The results revealed marked compositional differences: whey was higher in dimethyl sulfone, succinate, orotate, fumarate, and lactose (*p* < 0.05), whereas WPC-80 contained significantly higher levels of histidine, formate, glucose + glucose-6-phosphate, acetate, and choline (*p* < 0.05). Moreover, metabolites such as hippurate, valine, lactate + threonine, and uracil were exclusively found on whey and not in WPC-80, likely due to processing steps such as ultrafiltration. **Conclusions:** These findings highlight the metabolic distinctions introduced by WPC-80 processing from Parmigiano Reggiano whey and provide insights into the nutritional and functional characteristics of whey-derived products. Such knowledge can inform the design of innovative dairy ingredients and functional foods, with potential benefits for both industry applications and consumer health.

## 1. Introduction

Whey and whey-derived protein concentrates are increasingly valued in the realm of food science and nutrition due to their high protein content, unique amino acid composition, bioactive potential, and functional versatility [1]. Whey is the liquid fraction generated after casein and fat removal during cheese manufacturing, representing one of the largest by-products of the dairy industry, with global production estimated in the tens of millions of tons annually [2]. Whey contains significant amounts of nitrogen, primarily in the form of proteins and other nitrogenous compounds. When released untreated into the environment, this nitrogen contributes substantially to nutrient pollution, leading to eutrophication in water bodies. Specifically, whey exhibits high nitrogen concentrations, which, combined with its biochemical oxygen demand and phosphorus content, fosters excessive algal growth and oxygen depletion in aquatic ecosystems. This eutrophication can cause toxic effects on aquatic organisms and disrupt aquatic biodiversity. Historically, this effluent was considered an environmental burden because of its high organic load; however, advances in protein fractionation and purification technologies have converted whey into a high-value ingredient [3].

Whey proteins are a highly bioavailable source of essential amino acids, particularly the branched-chain amino acids (BCAAs) leucine, isoleucine, and valine, which play crucial roles in energy metabolism, neural function, and muscle protein synthesis [4]. Although raw whey is often used in animal feed or biogas generation, it can be refined into high-quality nutritional products such as whey protein, and its derivatives such as whey protein concentrates (WPCs) and isolates (WPIs). Compared with other dairy powders, WPCs and WPIs offer superior digestibility and bioavailability, partly due to their higher content of β-lactoglobulin, α-lactalbumin, bovine serum albumin, immunoglobulins, and lactoferrin [5]. These proteins, along with casein-derived peptides such as glycomacropeptide, contribute to whey’s function, from emulsification and foaming to bioactive health effects including the ability to inhibit bacterial and viral adhesion and modulate immune responses [6].

Increasing evidence supports the beneficial effects of whey protein supplementation on muscle health across different age groups. In older adults with sarcopenia (≥60 years), whey protein intake has been shown to significantly improve muscle mass and physical performance, with synergistic effects on strength when combined with resistance training [7]. Similarly, in a study of younger men (aged 20–30 years), four weeks of resistance training led to a significantly greater increase in muscle mass among participants supplemented with whey protein compared to those receiving a placebo, highlighting the ability of whey protein to enhance the anabolic response to exercise [8]. For children, the regular consumption of whey protein may support healthy growth and immune function by improving protein digestibility and providing bioactive peptides with potential anti-inflammatory effects, although excessive intake or highly processed forms could alter digestion and gut response [9].

The nutritional quality of whey powders is strongly influenced by the manufacturing route. Distinct processing steps such as ultrafiltration, hydrolysis, or demineralization alter the composition in terms of amino acids and non-protein nitrogen fractions [10]. Among these products, whey protein concentrate at 80% (WPC-80) represents a high-quality whey protein powder containing approximately 80% protein by weight, produced through filtration and drying processes that effectively remove water, lactose, and fat, yielding a nutrient-dense source of essential amino acids [11]. Due to its distinctive amino acid profile, WPC-80 differs substantially from whole milk powders or casein-containing fractions, with important implications for its use in sports nutrition, infant formula, and medical foods [5].

Beyond their nutritional role, whey proteins also serve as versatile ingredients, both in terms of their technological and functional properties. They can act as thickening or gelling agents, carriers for micronutrients and bioactives, and stabilizers in food matrices [4]. Their ability to interact with small molecules enhances solubility, bioaccessibility, and delivery efficiency, further reinforcing their role as sustainable and multifunctional food components. Therefore, understanding the metabolite composition of whey and WPC-80 is essential for evaluating their nutritional and functional properties. Proton nuclear magnetic resonance (^1^H NMR) spectroscopy enables detailed profiling of these compounds in dairy matrices, offering insights into their nutritional and technological attributes. Alongside mass spectrometry (MS), ^1^H NMR is one of the two main tools for metabolomic analysis [12]. Compared with MS, ^1^H NMR provides distinct advantages, including higher reproducibility, non-destructive measurement, and the ability to simultaneously identify and quantify a wide range of organic molecules, enabling precise structural characterization. Further benefits include simplified sample handling and compatibility with automated workflows, which enhance analytical efficiency. However, limitations such as relatively low sensitivity, spectral overlap, and challenges with reproducibility and standardization must be acknowledged. Despite these limitations, ^1^H NMR-based metabolite fingerprinting remains a rapid, practical, and reliable analytical tool, applied also for characterization and authentication of dairy products [13,14,15].

This study aimed to characterize and compare the metabolic profiles of whey and WPC-80 from the Parmigiano Reggiano production chain using ^1^H NMR spectroscopy, providing an evaluation of their metabolite profiles and contributing to a better understanding of the nutritional and functional properties of these derived dairy products.

## 2. Materials and Methods

### 2.1. Sample Preparation

Five whey samples and four WPC-80 powders were obtained from an Italian dairy company producing Parmigiano Reggiano (Albalat, Modena, Italy). Whey protein concentrate at 80% powders were suspended in ultrapure water. The dilution was adjusted until the average density of the WPC-80 solutions matched that of whey samples, as verified by refractometric analysis.

Sample treatment followed the procedure described in Tenori et al. [16]. Each sample was combined in equal proportion with dichloromethane (CH_2_Cl_2_) and vortexed to ensure homogenization. The mixtures were left at room temperature (25 °C) for 10 min to allow phase separation and were then centrifuged at 5000× *g* for 30 min at 4 °C. After extraction with dichloromethane, the upper aqueous phase, containing soluble polar metabolites, was carefully collected (350 µL). The protein layer at the interface and the organic CH_2_Cl_2_ phase were discarded. This protocol, efficiently removes lipids and macromolecules while preserving the polar metabolome, yielding clean and reproducible spectra. Thereafter, 350 μL of the upper aqueous phase were added to 350 μL of sodium phosphate buffer [70 mM Na_2_HPO_4_; 20% (vol/vol) H_2_O, 6.1 mM NaN_3_; 4.6 mM sodium trimethylsilyl (2,2,3,3-H4)-propionate; pH 7.4]. A total of 600 μL of the resulting mixture were transferred into a 5 mm ^1^H NMR tube and kept at −80 °C until further analysis at the Magnetic Resonance Center (Sesto Fiorentino, Florence, Italy).

### 2.2. Nuclear Magnetic Resonance

One-dimensional (1D) ^1^H NMR spectra were acquired at 600.13 MHz using a Bruker Avance spectrometer (Bruker, Rheinstetten, Germany) equipped with a 5 mm PATXI probe and an autosampler (SampleJet, Bruker, Rheinstetten, Germany).

Sample temperature was regulated at 37 °C with a high-precision thermocouple, and loading was managed with an automated autosampler.

For each sample, an ^1^H NMR spectrum was acquired using a nuclear overhauser effect spectroscopy (NOESY) presaturation pulse sequence (noesygppr1d). Acquisition parameters included 64 scans, 4 dummy scans, 98k data points, a spectral width of 18.028 kHz, an acquisition time of 2.7 s, a relaxation delay of 4 s, a mixing time of 10 ms, a pulse length of 10 µs, a receiver gain of 90.5, and a presaturation power of 1.35 × 10^−5^ W.

Post-processing involved exponential multiplication of the free induction decay with a 0.3 Hz line-broadening factor, Fourier transformation, and automatic correction for baseline and phase distortions. Spectral alignment was carried out using the α-lactose doublet at 5.24 ppm as a chemical shift reference.

The spectral region from 0.02–10.00 ppm was subdivided into 0.02-ppm buckets. Buckets corresponding to residual water (4.61–4.93 ppm) and dichloromethane signals (5.29–5.67 ppm) were removed. This procedure reduced each spectrum to 328 variables for statistical analysis. Metabolites assignment was performed using reference spectral libraries of compounds (Chenomx NMR Suite), FoodDB, and the Milk Composition Database, complemented by published reports [16,17,18]. Peak integration was performed using an in-house R (version 4.3.1, R Foundation for Statistical Computing, Vienna, Austria) script. Metabolite quantification in arbitrary units was performed through integration of representative, well-resolved peaks. In the case of partial signal overlap, only non-overlapping and well-defined resonances were considered for quantification, while signals with significant overlap were excluded to avoid introducing bias.

### 2.3. Statistical Analysis

Data analysis was conducted using Python (version 3.9, Wilmington, DE, USA). Probabilistic quotient normalization was applied to ensure comparability in all statistical analyses [19]. Differences in metabolic content between whey and WPC-80 samples were initially evaluated using the Mann–Whitney U test, after applying log_10_ transformation and autoscaling, as the data did not follow a normal distribution. Variation in metabolite content was expressed as logarithmic fold change (base 2).

Two types of multivariate analyses were conducted: principal component analysis (PCA) and partial least squares-discriminant analysis (PLS-DA). These were performed on two datasets: (i) one comprising the entire ^1^H NMR spectral data and (ii) one comprising the quantified metabolites. Principal component analysis served as the initial exploratory tool, providing an unsupervised overview of data patterns. Prior to analysis, the data were log_10_-transformed and autoscaled [20]. Partial least squares-discriminant analysis was employed as a supervised method to identify key metabolites distinguishing between groups and examine collective metabolite behaviors. Partial least squares-discriminant analysis models built using metabolite data were log_10_-transformed and autoscaled, while spectral data were only autoscaled. The PLS-DA models were validated using k-fold cross-validation, with k determined by sample size per group [21]. Variable importance in projection (VIP) scores were extracted, with metabolites considered significant for model prediction when VIP > 1.

To evaluate practical equivalence between Whey and WPC-80 for each metabolite, the two one-sided tests (TOST) procedure for independent samples with unequal variances (Welch) was applied. We set the significance level to α = 0.05 for each one-sided test. Because peak areas are in arbitrary units and differ in scale and variability across metabolites, we defined metabolite-specific (hybrid) equivalence margins as:

$${∆}_{i}=max(0.2\times \bar{x_{i}}\;,\;2\times {SD}_{within,\;whey,i})$$

where $\bar{x_{i}}$ is the overall mean of metabolite *i* (across both groups) and *SD_within,Whey,i_* is the within-group SD estimated in the reference group (whey). A minimal epsilon (1 × 10^−6^) prevented zero margins for near-zero signals. For each metabolite, non-finite values (NA/NaN/Inf) were removed; no imputation or log transformation was applied. A metabolite was declared equivalent only if both one-sided tests were significant (*p*_lower < α and *p*_upper < α) or if the (1 − 2α) = 90% confidence interval for the mean difference (Whey − WPC-80) lay entirely within [−Δi, +Δi] [22]. Given the limited number of samples available for this pilot study, we also performed an analysis-informed power estimation using the MetSizeR framework (R Foundation for Statistical Computing, Vienna, Austria) to quantify the statistical limitations associated with our design.

## 3. Results

### 3.1. Exploratory Data Analysis

**Figure 1:**
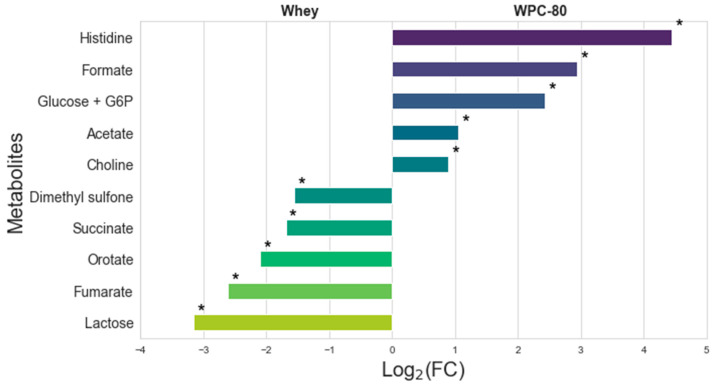
Logarithmic fold change [Log_2_(FC)] of the area under the peaks of the identified metabolites. Positive Log_2_(FC) bars indicate higher metabolite levels in whey protein concentrate containing 80% (WPC-80) compared to whey samples and negative Log_2_(FC) bars indicate lower metabolite levels in WPC-80 compared to whey samples. * *p* < 0.05.

A total of 30 metabolites were identified in whey and WPC-80 samples. Several metabolites were detected exclusively in whey, including 2-oxoglutarate, 2-oxoglutarate + carnitine, alanine, carnitine, citrate, ethanol, glycerophosphocholine, hippurate, isoleucine, lactate + threonine, N-acetyl carbohydrates, O-acetylcarnitina, phosphocreatine + creatine, phenylalanine, trans-aconitate, tyrosine, uracil, and valine (i.e., they were absent in WPC-80: Table 1). Whey and WPC-80 showed notable metabolic differences (Figure 1). Based on the Mann–Whitney U test, levels of histidine, formate, glucose + glucose-6-phosphate, acetate, and choline were significantly greater in WPC-80 compared to whey (*p* < 0.05). Conversely, dimethyl sulfone, succinate, orotate, fumarate, and lactose levels were greater in whey compared to WPC-80 (*p* < 0.05; Table S1).

### 3.2. Principal Component Analysis

**Figure 2:**
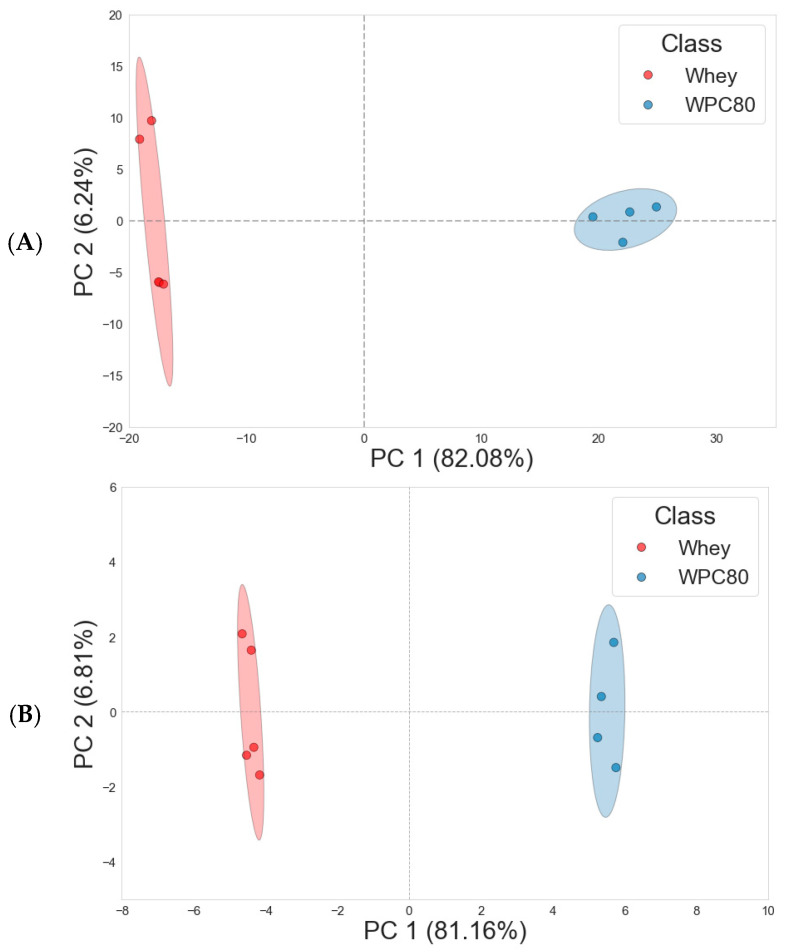
Score plot of first (PC1) and second (PC2) principal components of the principal component analysis (PCA) of whey and whey protein concentrate at 80% (WPC-80): (**A**) PCA performed on ^1^H NMR bucketed spectra; (**B**) PCA performed on relative quantification of metabolites. The ellipse represents the 95% confidence interval. Each dot in the plots represents a sample, with red indicating whey samples and blue indicating WPC-80 samples.

Examination of the PCA results from both bucketed spectra and metabolic content revealed no evidence of outliers in either dataset, as illustrated in Figure 2. The analysis demonstrated distinct clustering among the two groups of samples, indicating substantial differences in their metabolic patterns. For the PCA conducted on the ^1^H NMR spectral buckets, the first principal component (PC1) accounted for 82.08% of the total variance and effectively discriminated between whey and WPC-80 samples. The second principal component (PC2) explained an additional 6.24% of the variance and further subdivided each of the whey and WPC-80 groups into two distinct clusters (Figure 2A). Parallel to the spectral analysis, the PCA performed on the quantified metabolic data showed that PC1 accounted for 81.16% of the total variance, while PC2 explained 6.81% (Figure 2B). Principal component analysis loadings derived from the quantified metabolomic data are presented in Figure S1, highlighting the variables that contribute most strongly to the separation.

### 3.3. Partial Least Squares-Discriminant Analysis

**Figure 3:**
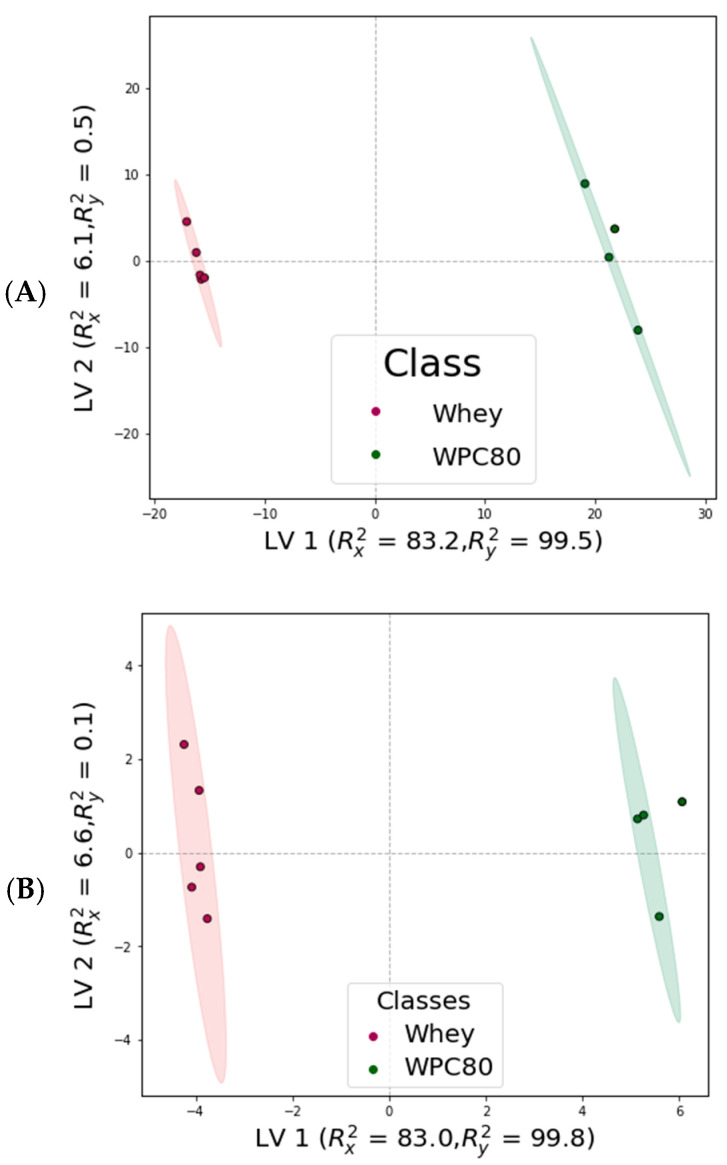
Score plot of partial least squares-discriminant analysis (PLS-DA) of whey and whey protein concentrate at 80% (WPC-80): (**A**) PLS-DA of ^1^H NMR bucketed spectra; (**B**) PLS-DA of ^1^H NMR metabolites. LV = latent variable (component). $R_{x}^{2}$ = fraction of variance in X. $R_{y}^{2}$ = fraction of variance in Y. Each dot in the plots represents a sample, with red indicating whey samples and green indicating WPC-80 samples.

The PLS-DA scores plot constructed using both the bucketed ^1^H NMR spectra and metabolic data demonstrated a distinct separation between whey and WPC-80 samples, confirming that their metabolic profiles differ significantly (Figure 3). For both metabolite and spectral datasets, the PLS-DA models yielded an accuracy and F1-score of 1.0, and a Q^2^ value of 0.99, indicating excellent predictive performance. The permutation test resulted in a pronounced reduction in model performance upon random shuffling of class labels, supporting that the classifier captures genuine class structure. Among the metabolites present in both the samples, lactose, histidine, and formate had the highest VIP scores indicating that they contributed most strongly to the differentiation between the two groups (Table 2). These results were consistent with the Mann–Whitney U test, which confirmed significant differences in histidine and formate content in WPC-80 and whey.

### 3.4. Equivalence Testing

Using TOST with the hybrid, metabolite-specific margins, 1/30 metabolites met the criteria for equivalence (Galactose), while 29/30 showed non-equivalent (practically relevant) differences between whey and WPC-80 (Table S1). Directionally, several compounds were higher in WPC-80 (negative difference, whey − WPC-80 < 0), notably choline, histidine, cis-aconitate, formate, glucose + glucose-6-phosphate, and acetate, whereas many others were higher in whey (positive difference, whey − WPC-80 > 0), including succinate, orotate, hippurate, dimethylsulfone, valine, 2-oxoglutarate + carnitine, carnitine, and uracil (Table S1). These equivalence results are consistent with the univariate and multivariate findings, supporting a systematic compositional shift associated with WPC-80 processing, with Galactose emerging as the only stable molecule under the predefined practical margins. Full TOST outputs (group means, differences, 90% CIs, margins, and *p*-values) are reported in the Table S2.

## 4. Discussion

Many studies have investigated the protein and amino acid composition of whey and WPC products [2,9], yet comparatively few have examined their broader metabolic profiles. Zacometti et al. [23] characterized metabolites in whey, microparticulated whey, and fermented microparticulated whey, highlighting the relevance of metabolomic approaches in dairy processing. In the present study, we applied a comprehensive metabolomic strategy to investigate the metabolic composition of whey and WPC-80 derived from the same production pipeline, allowing a more detailed assessment of compositional changes across processing steps. Proton nuclear magnetic resonance spectroscopy was used in combination with two complementary strategies: bucketed spectral analysis (providing a broad overview of whey and WPC-80 metabolic patterns), and targeted metabolite quantification (identifying specific compounds driving differences between groups). Together, these methods enabled robust characterization of whey and WPC-80. However, we acknowledge that the study is based on a limited sample size (five whey and four WPC-80 samples), which constrains statistical power. To formally evaluate the implications of this reduced sample size, we performed an analysis-informed power assessment using the MetSizeR framework, which is specifically designed for metabolomic datasets. Based on our bucketed spectra (328 variables), targeted metabolites (30 variables), a 20% expected proportion of significant features, PPCA as the planned analytical method, and a target FDR of 0.05, MetSizeR estimated that approximately 28 samples (≈14 per group) would be required for the bucket dataset and 22 samples (≈11 per group) for the targeted metabolites to achieve adequate statistical power. Our current sample size was therefore underpowered, and the results must be interpreted as preliminary and exploratory. Following this evaluation, we carefully avoided strong inferential claims and considered the findings as hypothesis-generating. This represents an inherent limitation but also an opportunity for future studies to validate these findings across multiple production batches, processing conditions and raw material sources.

### 4.1. Differences in Metabolite Abundance Between Whey and WPC-80

The comparative analysis of whey and WPC-80 revealed pronounced differences in their metabolite profiles (Figure 1, Figure 2 and Figure 3). The metabolites that were present in WPC-80, such as acetate, choline, cis-aconitate, dimethyl sulfone, formate, fumarate, galactose, glucose + glucose-6-phosphate, histidine, lactose, orotate, and succinate, are primarily polar or multicharged organic acids and sugars. Their capacity to form electrostatic or hydrogen-bond interactions with whey proteins and minerals increases their apparent molecular size, which hinders their diffusion through the ultrafiltration membrane pores during the ultrafiltration process. This interaction with proteins and minerals effectively prevents these metabolites from passing into the permeate, causing them to be retained in the retentate fraction [24]. In contrast, metabolites that passed into the permeate, present in Whey, such as 2-oxoglutarate, alanine, carnitine, citrate, ethanol, glycerophosphocholine, hippurate, isoleucine, lactate + threonine, N-acetyl carbohydrates, phosphocreatine + creatine, phenylalanine, trans-aconitate, tyrosine, uracil, and valine, are generally smaller, neutral, or weakly interacting compounds. These properties enable them to remain freely dissolved in the aqueous phase and easily diffuse through the membrane pores during ultrafiltration. However, the intrinsic characteristics of the ultrafiltration process not only reduce the overall protein recovery efficiency but also adversely affect lactose recovery and purification [25]. Overall, the retention or passage of these metabolites during ultrafiltration depends on their molecular size, charge, polarity, and interaction strength with whey proteins and minerals, which influence their apparent molecular size and membrane permeability.

The identified amino acids are nutritionally and functionally important, with BCAAs such as valine and isoleucine playing key roles in protein synthesis, muscle maintenance, neuronal activity, glucose homeostasis, and overall metabolic regulation [1,4,26]. Aromatic amino acids such as tyrosine and phenylalanine were also present only in whey. Tyrosine serves as the direct precursor of catecholamine neurotransmitters, including dopamine, norepinephrine, and epinephrine, and its availability can influence their rate of synthesis. Phenylalanine, which is hydroxylated to form tyrosine, represents the initial and rate-limiting precursor in catecholamine biosynthesis. Together, these amino acids play a crucial role in regulating the production of key neurotransmitters, highlighting their potential neurological significance [27]. Although the free amino acid fraction is reduced or absent in WPC-80, the higher protein concentration compensates for this, since dietary proteins are hydrolyzed during digestion to release amino acid residues that contribute to the same metabolic pool. Whey protein concentrate at 80% contains concentrated intact proteins, which are polymers of amino acid residues. During digestion, these proteins are enzymatically hydrolyzed, releasing the constituent amino acids, including those that were initially absent in free form. This allows for efficient absorption and metabolism of essential amino acids even from WPC-80, maintaining its nutritional value despite the reduced free amino acid content [28].

Citrate was another metabolite detected exclusively in whey. As a major component of milk serum, citrate regulates mineral balance by buffering calcium and hydrogen ions, forming soluble complexes with calcium, and preventing calcium phosphate precipitation [29,30,31]. This citrate-induced redistribution of calcium has important effects on milk stability, structure, and technological properties. Furthermore, citrate can interact with whey proteins under specific conditions, modifying their structure. For instance, the addition of citric acid to whey protein isolate has been shown to improve emulsifying and foaming properties, thereby enhancing its performance in food systems requiring these functionalities [32].

In contrast, WPC-80 presented a higher content in histidine, formate, glucose (including glucose-6-phosphate), acetate, and choline (*p* < 0.05), likely reflecting a concentration effect from ultrafiltration. Histidine, an essential amino acid participating in protein synthesis and enzymatic activity, was found in higher abundance in WPC-80, together with other energy-related metabolites such as glucose and glucose-6-phosphate [33]. Elevated histidine levels may enhance the nutritional quality of WPC-80, supporting muscle protein turnover and contributing to buffering capacity, an aspect relevant for both metabolic health and the formulation stability of high-protein products. Choline, which supports cell membrane integrity and neurotransmission, was also elevated in WPC-80, contributing to its nutritional value, particularly for brain health. Its increased presence enhances the nutritional profile of the ingredient, particularly with regard to cognitive development and liver function [34].

Conversely, whey contained higher levels of dimethyl sulfone, succinate, orotate, fumarate, and lactose; the latter’s reduction in WPC-80 is consistent with its formulation as a high-protein, reduced-lactose ingredient suitable for lactose-intolerant consumers. Moreover, based on Niero et al. [35], the following metabolites quantified in our study: acetate, lactose, glucose, galactose, succinate, fumarate, orotate, valine, hippurate, dimethyl sulfone, lactate, carnitine, phosphocreatine + creatine, 2-oxoglutarate, cis-aconitate, and citrate, were also identified in individual milk samples and were used to evaluate grazing effects, highlighting the possible role of different metabolites as markers of farm management.

Taken together, results of the present study demonstrate that processing steps such as ultrafiltration substantially alter the metabolite composition of dairy products. While whey retains a diverse array of low-molecular-weight amino acids and organic acids, WPC-80 is richer in protein-associated and energy-related metabolites. The drying and subsequent rehydration of WPC-80 may also influence its metabolic profile, for example, through compound degradation, volatilization, or differential solubility. These aspects warrant deeper investigation in future studies to fully clarify their contribution to the metabolic differences observed.

The compositional shifts from whey and WPC-80 metabolic composition influence not only the nutritional value of the products but also their technological and functional properties, with implications for food formulation and the nutraceutical industry.

### 4.2. PCA-Based Clustering and Key Metabolites Identified by PLS-DA in Whey and WPC-80

Multivariate analyses revealed a clear separation between whey and WPC-80. Principal component analysis showed strong clustering, with the first two components explaining over 87% of the total variance in both spectral and metabolite datasets. Consistently, the PCA based on quantified metabolic data indicated that PC1 and PC2 reflected the trends observed in the spectral analysis (Figure 2).

Partial least squares-discriminant analysis identified lactose, histidine, and formate as the main discriminant metabolites, showing the highest VIP scores (Table 2). These compounds not only distinguish whey from WPC-80 but also reflect changes resulting from processing. This integrated analytical approach allowed a reliable identification of key metabolic differences and trends among the sample groups. The consistent significance of these metabolites across different statistical analyses indicates that they may serve as potential markers for monitoring the efficiency and reproducibility of WPC-80 production.

### 4.3. Practical Relevance via Equivalence Testing

To gauge practical relevance, we applied TOST with hybrid, metabolite-specific margins to accommodate unequal variances with a small number of samples. This makes the margin both scale-aware and anchored to analytical variability. Only galactose met the equivalence criteria (1/30); 29/30 metabolites were non-equivalent, in line with univariate/multivariate results. Directionally, WPC-80 > whey for choline, histidine, cis-aconitate, formate, glucose + glucose-6-phosphate, and acetate, while whey > WPC-80 for lactose, succinate, orotate, hippurate, valine, and carnitine.

## 5. Conclusions

The absence of certain metabolites in WPC-80 may serve as a distinguishing feature of the protein concentration process. It should also be noted that variations in processing methods can lead to distinct compositional outcomes; therefore, a more complete understanding will require additional studies involving diverse concentration techniques and whey sources.

By integrating both spectral and targeted metabolite analyses, this study provides a detailed characterization of whey and WPC-80. Whey protein concentrate at 80% showed significantly higher levels of histidine, formate, glucose, glucose-6-phosphate, acetate, and choline (*p* < 0.05), whereas whey contained higher amounts of dimethyl sulfone, succinate, orotate, fumarate, and lactose (*p* < 0.05). Among the discriminant metabolites, lactose, histidine, and formate had the highest VIP scores. Using TOST with hybrid, metabolite-specific margins, only galactose met equivalence criteria (1/30), while 29/30 metabolites showed non-equivalent, practically relevant differences. The inclusion of TOST equivalence testing allows us to formally assess when metabolite levels are not only statistically non-different, but statistically equivalent within biologically meaningful limits.

These findings underscore how processing shapes the metabolite composition of whey-derived products, particularly within the Parmigiano Reggiano production chain, and demonstrate the value of ^1^H NMR for detailed compositional assessment. Given the small number of biological replicates available, these findings should be regarded as preliminary and require confirmation in larger, adequately powered studies. The resulting dataset provides a useful reference framework that may support future investigations in other dairy systems and guide the development of functional foods and optimized whey-derived products.

## Figures and Tables

**Table 1:** Descriptive statistics for metabolic composition of whey and whey protein concentrate at 80% (WPC-80).

Metabolite (× 10^−4^)	Whey (n = 5)					WPC-80 (n = 4)			
	Mean	Minimum	Maximum	CV, %		Mean	Minimum	Maximum	CV, %
2-Oxoglutarate	0.53	0.457	0.618	11.79		-	-	-	-
2-Oxoglutarate + Carnitine	1.96	1.7	2.2	13.28		-	-	-	-
Acetate	9.39	4.17	17.17	75.28		19.54	19.30	19.90	1.58
Alanine	0.5	0.48	0.52	3.22		-	-	-	-
Carnitine	0.139	0.081	0.188	29.33		-	-	-	-
Choline	5.72	3.45	7.56	34.09		10.64	8.29	12.99	25.20
Cis-aconitate	0.18	0.068	0.25	41.93		0.31	0.13	0.51	57.70
Citrate	43.37	37.23	47.79	9.97		-	-	-	-
Dimethyl sulfone	0.87	0.59	1.09	29.78		0.30	0.25	0.34	14.35
Ethanol	0.31	0.080	0.52	51.55		-	-	-	-
Formate	0.15	0.13	0.17	10.77		1.13	1.06	1.21	5.88
Fumarate	0.20	0.12	0.33	53.88		0.03	0	0.07	115.94
Galactose	10.59	10.35	10.88	2.32		10.33	9.23	11.64	12.01
Glucose + Glucose-6-Phosphate	2.74	1.30	3.74	46.67		14.80	12.60	17.08	16.58
Glycerophosphocholine	38.80	35.65	40.98	7.22		-	-	-	-
Hippurate	1.05	0.73	1.49	37.52		-	-	-	-
Histidine	0.23	0.21	0.26	9.17		5.01	4.25	6.03	16.71
Isoleucine	0.022	0	0.044	94.35		-	-	-	-
Lactate + Threonine	127.39	98.58	168.64	28.07		-	-	-	-
Lactose	616.88	613.46	621.09	0.54		69.11	59.83	77.42	13.28
N-Acetyl carbohydrates	28.57	24.34	31.82	13.30		-	-	-	-
O-Acetylcarnitina	0.58	0.35	0.92	51.33		-	-	-	-
Orotate	1.97	1.86	2.08	4.45		0.46	0.37	0.59	21.52
Phosphocreatine + Creatine	10.58	10.19	10.89	2.95		-	-	-	-
Phenylalanine	0.046	0.018	0.068	44.20		-	-	-	-
Succinate	3.19	3.08	3.32	3.50		0.99	-	1.94	109.04
Trans-aconitate	0.07	0.026	0.11	50.80		-	-	-	-
Tyrosine	0.059	0	0.103	67.44		-	-	-	-
Uracil	0.066	0	0.091	57.17		-	-	-	-
Valine	0.21	0.189	0.24	9.25		-	-	-	-

**Table 2:** Discriminatory traits based on variable importance in projection ≥ 1 identified in the partial least squares-discriminant analysis (PLS-DA) and respective latent variables (LV) 1 and 2.

Metabolite	VIP	LV1	LV2	Metabolite	VIP	LV1	LV2
2-Oxoglutarate	1.104	−0.20	−0.02	Hippurate	1.103	−0.20	−0.01
2-Oxoglutarate and Carnitine	1.104	−0.20	−0.02	Histidine	1.102	0.20	0.02
Acetate	0.748	0.14	0.30	Isoleucine	0.700	−0.13	−0.19
Alanine	1.105	−0.20	−0.03	Lactate + Threonine	1.104	−0.20	−0.02
Carnitine	1.103	−0.20	−0.06	Lactose	1.102	−0.20	−0.03
Choline	0.830	0.15	−0.18	N-Acetyl carbohydrates	1.104	−0.20	−0.04
Cis-aconitate	0.484	0.09	−0.39	O-Acetylcarnitine	1.101	−0.20	0.00
Citrate	1.105	−0.20	−0.03	Orotate	1.091	−0.20	0.00
Dimethylsulfone	1.008	−0.18	−0.23	PCr + Creatine	1.105	−0.20	−0.03
Ethanol	1.097	−0.20	0.02	Phenylalanine	1.096	−0.20	−0.05
Formate	1.102	0.20	0.05	Succinate	0.684	−0.12	0.11
Fumarate	0.793	−0.14	0.41	Trans-aconitate	1.097	−0.20	−0.02
Galactose	0.275	−0.04	−0.01	Tyrosine	0.891	−0.16	0.18
Glucose + G6P	1.021	0.19	−0.10	Uracil	0.880	−0.16	0.02
Glycerophosphocholine	1.105	−0.20	−0.03	Valine	1.104	−0.20	−0.04

## Data Availability

The data presented in this study are available upon request from the corresponding or the first author due to privacy reasons.

## Supplementary Materials

The following supporting information can be downloaded at https://www.mdpi.com/article/10.3390/metabo15120770/s1: Table S1: *p*-values from the Mann–Whitney U test and 95% confidence intervals for the fold-change analysis. Table S2: Full TOST (Welch; α = 0.05) results for all metabolites, with hybrid margins per metabolite. Figure S1: Loading plot of the principal component analysis (PCA) performed on the relative quantification of metabolites from whey and whey protein concentrate (WPC-80).

## Abbreviations

The following abbreviations are used in this manuscript:

1D	One-dimensional
^1^H NMR	Proton nuclear magnetic resonance
BCAAs	Branched-chain amino acids
LV	Latent variable
MS	Mass spectrometry
NOESY	Nuclear Overhauser Effect Spectroscopy
PCA	Principal component analysis
PC	Principal component
PLS-DA	Partial least squares discriminant analysis
TOST	Two One-Sided Tests
VIP	Variable Importance in Projection
WPC	Whey protein concentrate
WPC-80	Whey protein concentrate at 80%
WPI	Whey protein isolate
