# Supplementary material for: 1H NMR for Comparative Metabolic Analysis of Whey and WPC-80
Source: Metabolites. 2025 Nov 28;15(12):770. doi: 10.3390/metabo15120770 (PMC12735035; doi:10.3390/metabo15120770)

Supplementary Table S1. P-values from the Mann–Whitney U test and 95% confidence intervals (95% CI) for the fold-change analysis.

Metabolite	p-value	95% CI (lower, upper)
Lactose	0.015873	-3.34, -3.08
Fumarate	0.019451	-3.67,-1.71
Orotate	0.015873	-2.34, -1.83
Succinate	0.015873	-6.82, -0.97
Dimethyl sulfone	0.015873	-1.83, -1.05
Galactose	1	-0.19, 0.06
Cis-aconitate	0.555556	-0.11, 1.45
Choline	0.015873	0.28, 1.30
Acetate	0.015873	0.41, 2.22
Glucose + G6P	0.015873	2.02, 2.95
Formate	0.015873	2.79, 3.06
Histidine	0.015873	4.23, 4.70

Supplementary Table S2. Full TOST (Welch; $\alpha = 0.05$) results for all metabolites, with hybrid margins per metabolite. Columns: Metabolite; Mean W (Whey); Mean whey protein concentrate at 80% (WPC-80); Difference W − WPC; 90% CI; ±Δ margin; p_{lower} ; p_{upper}; Equivalent (Yes/No).

Metabolite	Mean W	Mean WPC-80	W-WPC	90%CI	±Δ	p_lower	p_upper	Equivalent
Succinate	3.19E+11	9.86E+10	2.20E+12	9.43E+10, 3.47E+11	4.42E+11	0.007804	0.97708	FALSE
Dimethylsulfone	8.71E+09	2.97E+09	5.75E+11	3.28E+10, 8.22E+10	5.19E+10	2.76E+11	0.670086	FALSE
Lactose	0.061688	0.006911	0.054776	0.053717, 0.055836	0.007469	4.52E+08	1	FALSE
Hippurate	1.05E+12	0	1.05E+12	6.77E+10, 1.43E+12	7.91E+10	2.39E+12	0.894405	FALSE
Valine	2.10E+11	0	2.10E+11	1.91E+10, 2.28E+11	3.88E+09	4.42E+09	0.99998	FALSE
Lactate+Threonine	0.012739	0	0.012739	0.00933, 0.016148	0.007152	1.20E+12	0.987475	FALSE
Acetate	9.39E+11	0.001954	-0.00101	-0.00169, -3.40E+11	0.001414	0.137354	7.62E+10	FALSE
Choline	5.72E+11	0.001064	-4.92E+11	-8.10E+11, -1.74E+12	3.90E+11	0.725166	0.001078	FALSE
Glucose+G6P	2.74E+11	0.00148	-0.00121	-0.00149, -9.22E+11	2.56E+11	0.999169	1.42E+11	FALSE
Galactose	0.00106	0.001033	2.69E+11	-1.18E+12, 1.72E+11	2.10E+12	0.01486	0.029047	TRUE
Cis-aconitate	1.79E+10	3.12E+10	-1.33E+11	-3.41E+09, 7.41E+09	1.50E+11	0.43558	0.022163	FALSE
Uracil	6.56E+10	0	6.56E+10	2.98E+10, 1.01E+11	7.50E+09	5.54E+11	0.302323	FALSE
Orotate	1.97E+11	4.60E+11	1.51E+12	1.39E+11, 1.63E+11	2.60E+10	5.14E+07	1	FALSE
Fumarate	2.03E+10	3.31E+08	1.70E+10	6.46E+10, 2.75E+11	2.18E+11	3.09E+11	0.196575	FALSE
Trans-aconitate	7.00E+09	0	7.00E+09	3.61E+09, 1.04E+11	7.11E+09	4.45E+12	0.473702	FALSE
Histidine	2.29E+10	5.01E+11	-4.78E+11	-5.77E+11, -3.80E+12	4.71E+11	0.999026	5.44E+11	FALSE
Formate	1.48E+10	1.13E+10	-9.82E+10	-1.06E+12, -9.05E+09	1.17E+11	0.999965	1.61E+08	FALSE
Isoleucine	2.16E+10	0	2.16E+10	2.17E+09, 4.10E+09	4.07E+10	0.001194	0.051706	FALSE
Ethanol	3.07E+09	0	3.07E+09	1.56E+10, 4.58E+10	3.17E+10	4.58E+11	0.449841	FALSE
Alanine	5.03E+09	0	5.03E+09	4.88E+10, 5.19E+11	5.59E+09	8.48E+07	1	FALSE
Citrate	0.004337	0	0.004337	0.003925, 0.004749	8.64E+09	5.67E+09	0.999972	FALSE
Tyrosine	5.92E+09	0	5.92E+09	2.11E+09, 9.73E+09	7.99E+09	7.33E+11	0.155967	FALSE
Phenylalanine	4.62E+09	0	4.62E+09	2.68E+10, 6.57E+09	4.09E+09	3.38E+12	0.705724	FALSE
N-Acetyl carbohydrates	0.002857	0	0.002857	0.002495, 0.003219	7.60E+11	1.44E+11	0.999876	FALSE
O-Acetylcarnitina	5.82E+10	0	5.82E+10	2.97E+11, 8.67E+10	5.98E+10	4.54E+11	0.456789	FALSE
2-Oxoglutarate+Carnitine	1.96E+11	0	1.96E+11	1.71E+11, 2.21E+11	5.21E+11	1.43E+11	0.999877	FALSE
Carnitine	1.39E+11	0	1.39E+11	1.00E+08, 1.78E+11	8.16E+09	1.34E+12	0.982777	FALSE
2-Oxoglutarate	5.25E+09	0	5.25E+09	4.66E+10, 5.84E+10	1.24E+11	9.82E+09	0.999934	FALSE
PCr+Creatine	0.001058	0	0.001058	0.001028, 0.001088	1.18E+12	5.98E+07	1	FALSE
Glycerophosphocholine	0.00388	0	0.00388	0.003612, 0.004147	5.60E+11	1.89E+10	0.999994	FALSE

Supplementary Figure S1. Loading plot of the principal component analysis (PCA) performed on the relative quantification of metabolites from whey and whey protein concentrate (WPC-80).

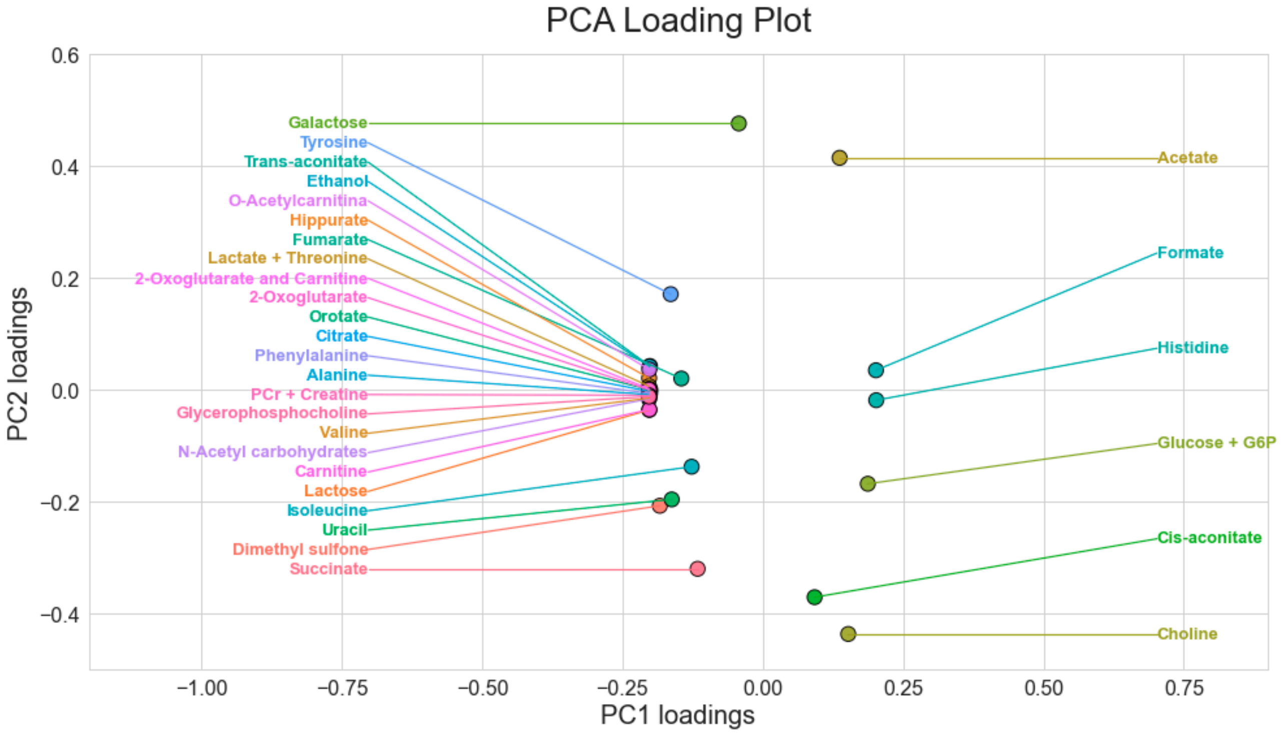

Supplement: Supplementary file 1 [file metabolites-15-00770-s001.zip › metabolites-3982715-supplementary.pdf]
